# Combined tracheostomy and maxillomandibular advancement for extremely severe obstructive sleep apnea: A case report

**DOI:** 10.1016/j.jobcr.2025.01.028

**Published:** 2025-02-11

**Authors:** Ning Zhou, Jean-Pierre T.F. Ho, Jan de Lange

**Affiliations:** a Department of Oral and Maxillofacial Surgery, Amsterdam University Medical Centers (UMC), University of Amsterdam, Meibergdreef 9, 1105 AZ, Amsterdam, the Netherlands; b Academic Centre for Dentistry Amsterdam (ACTA), University of Amsterdam and Vrije Universiteit Amsterdam, 1081 LA, Amsterdam, the Netherlands; c Department of Oral and Maxillofacial Surgery, Northwest Clinics, 1815 JD, Alkmaar, the Netherlands

**Keywords:** Obstructive sleep apnea, Tracheostomy, Maxillomandibular advancement

## Abstract

Tracheostomy and maxillomandibular advancement (MMA) are both highly effective surgical treatments for adult obstructive sleep apnea (OSA). The present case study described the successful application of combined tracheostomy and MMA for the treatment of extremely severe OSA.

## 1 Introduction

Obstructive sleep apnea (OSA) is a common sleep-related breathing disorder.^1^ Continuous positive airway pressure (CPAP) is the current gold standard therapy for moderate to severe OSA. Nevertheless, the acceptance and adherence of CPAP remains a challenge in quite a few patients, which suggests the necessity of alternative treatments.^2^

Prior to the advent of CPAP, tracheostomy was used as the standard of care for OSA patients, especially severe OSA patients. Current use of tracheostomy in OSA serves either as a permanent therapy in highly selected cases not amenable to other therapies, or as a temporary procedure for airway protection in patients who have high risk of airway compromise in the perioperative period of other OSA surgical procedures.^3^^,^^4^ Aside from tracheostomy, maxillomandibular advancement (MMA) is currently considered the most effective surgery for OSA.^5^

The purpose of this case study was to report a successful case using temporary tracheostomy and MMA in a patient with extremely severe OSA and CPAP intolerance.

## 2 Case report

**Fig. 1:**
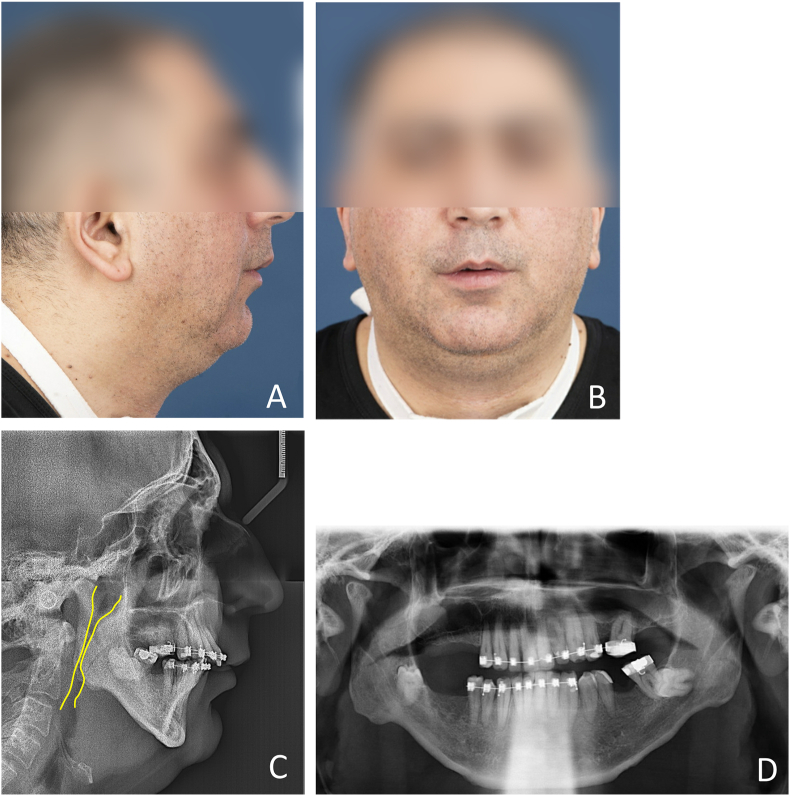
Before maxillomandibular advancement surgery. A, profile view of the extra-oral photograph; B, frontal view of the extra-oral photograph; C, lateral cephalogram; D, panoramic radiograph.

A 40-year-old male was referred to the Department of Oral and Maxillofacial Surgery for MMA surgery consultation. He was diagnosed with extremely severe OSA as confirmed by an overnight polysomnography (apnea hypopnea index [AHI] = 107 events/hour) and was severely obese (body mass index [BMI] = 35 kg/m^2^). The oxygen desaturation index of 4 % (ODI 4 %) was 88 events/hour. The patient presented severe subjective symptoms. He has failed CPAP therapy and positional therapy. Otorhinolaryngology (ENT） examinations revealed nasal concha hypertrophy and a bulky tongue, due to which only the hard palate was visible (Friedman tongue position class 4 and modified Mallampati score of 4). During drug-induced sleep endoscopy (DISE) in supine position, a complete concentric collapse at the soft palate and a complete lateral collapse at the oropharynx were observed; the tongue base and epiglottis were not visible. The jaw thrust maneuver performed in the supine position showed a mild effect in improving upper airway obstruction. Radiological and dental examinations revealed a severe mandibular retrognathia, accompanied by a mutilated dentition with Class II division 1 malocclusion (Fig. 1). A treatment plan was made by the oral and maxillofacial surgeon and an orthodontist, which included tracheostomy, presurgical orthodontic preparation, MMA surgery, decannulation, and post-surgical orthodontics.

One month prior to the presurgical orthodontic preparation, the patient underwent a Bjork flap tracheostomy under general anesthesia. The purpose of tracheostomy was to present immediate OSA relief to the patient while receiving presurgical orthodontics decompensation. Immediately the patient looked more rested, and he reported that his overall condition and sleep were greatly improved after tracheostomy.

Seven months after tracheostomy, the presurgical orthodontics was completed. Computer-assisted three-dimensional surgical planning was performed one week prior to MMA surgery.

**Fig. 2:**
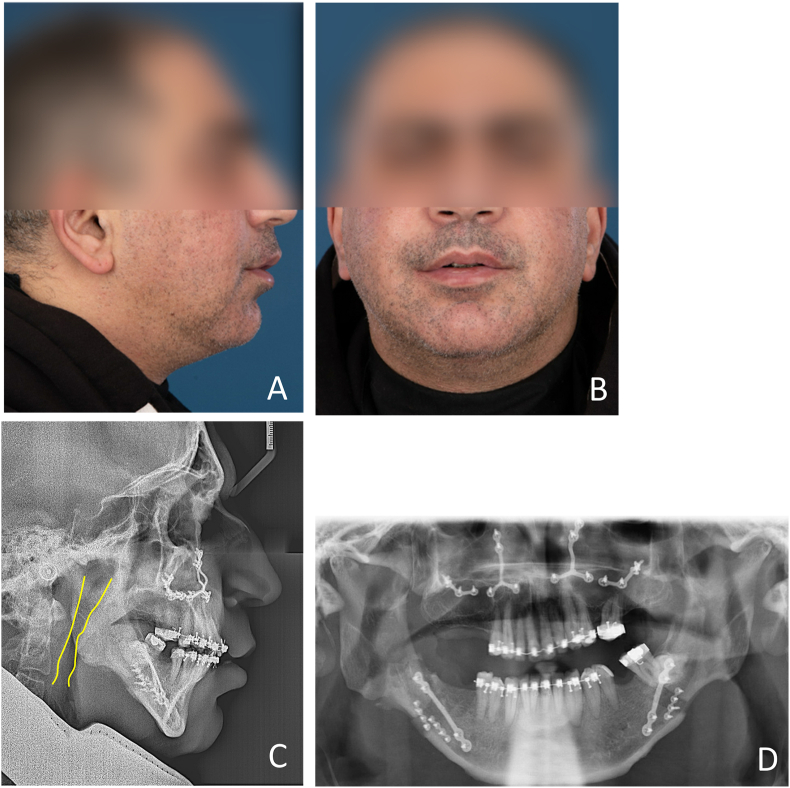
After maxillomandibular advancement surgery. A, profile view of the extra-oral photograph; B, frontal view of the extra-oral photograph; C, lateral cephalogram; D, panoramic radiograph.

Eight months after tracheostomy, the patient underwent MMA surgery under general anesthesia (Fig. 2). The degrees of maxillary and mandibular advancement were 7.8 mm and 10.9 mm, respectively. The postoperative course was uneventful.

Postoperatively, the plan was to perform decannulation after assessing the patient's sleeping condition with cannula capping, through an overnight PSG recording. Seven months after MMA the patient wished to have the tracheostomy removed without prior PSG examination, due to personal reasons and the fact that he was experiencing more irritation of stoma. Decannulation was performed under local anesthesia. The airway maintained patent and there was no stridor, decrease in oxygen saturation, or signs of respiratory distress after decannulation.

Nine-months after MMA, a follow-up PSG was performed. The PSG report suggested an almost complete cure of the patient's OSA with a postoperative AHI of 5.1 events/hour. The lowest oxygen saturation and ODI 4 % was 89 % and 3.8 events/hour, respectively.

At the last follow-up (one year after MMA), physical examination showed that tracheostomy stoma healed well. Regular appointments were arranged for long-term follow-up.

## 3 Discussion

The popularization of many alternative treatment modalities for OSA, such as CPAP therapy, has reduced the need for a tracheostomy in OSA treatment. Although tracheostomy is now considered a historical or outdated intervention for the treatment of OSA, it remains significant clinical value in highly selected cases where it serves as a life-saving or temporizing intervention to ensure airway patency in severe or refractory OSA. This case report illustrates the successful use of combined tracheostomy and MMA in a patient with severe OSA and CPAP intolerance.

For patients undergoing MMA for OSA, presurgical orthodontics may produce a better postsurgical occlusion, but it may also exaggerate the malocclusion and make the skeletal deformity more noticeable at the same time.^6^ As the patient described in this case report presented considerable retrognathia, a risk factor of OSA,^7^ it was contemplated that presurgical decompensation might further aggravate the patient's OSA symptoms. Considering that, the treatment team decided to offer temporary tracheostomy for airway protection in the period of presurgical orthodontics.

In addition to the aforementioned treatment options for OSA, multiple alternative therapies are available, mainly including oral appliance therapy, positional therapy, upper airway surgery, and hypoglossal nerve stimulation (HNS).^8^. Oral appliances, such as mandibular advancement devices (MADs), are particularly indicated for patients with mild to moderate OSA. MADs may also serve as an alternative for patients with severe OSA who do not tolerate CPAP therapy, although their efficacy is generally lower.^9^. Positional therapy, a behavioral intervention, is typically indicated for patients with positional OSA.^10^. Upper airway surgeries are often considered for patients with anatomical risk factors contributing to OSA, such as tonsillar hypertrophy and macroglossia.^11^. HNS has emerged as a promising alternative treatment for moderate to severe OSA in carefully selected patients. According to the U.S. Food and Drug Administration, the indication criteria of HNS include the following: adults older than 18 years of age; diagnosed with moderate to severe OSA (AHI 15–100 events/hour) (of which less than 25 percent of events are central and mixed apneas); failure or intolerance of CPAP therapy; BMI less than 40 kg/m^2^; and absence of complete concentric collapse at the soft palate.^12^.

While the patient in this case benefited from the combination of temporary tracheostomy and MMA, alternative therapies such as those mentioned above may be considered for other patients based on individual characteristics, clinical indications, and patient preference. This highlights the importance of a personalized approach in OSA management.

## 4 Conclusion

In conclusion, this case report shows that in highly selected patients, the combination of tracheostomy and MMA is an effective and relatively safe treatment option for severe OSA. Specifically, for OSA patients who are intolerant of CPAP and require airway protection during presurgical orthodontic treatment, tracheostomy can be an effective option.

## Ethical clearance

Not applicable.

## Consent

Informed consent was obtained from the patient.

## Sources of funding

This research did not receive any specific grant from funding agencies in the public, commercial, or not-for-profit sectors.

## Declaration of competing interest

The authors declare that they have no known competing financial interests or personal relationships that could have appeared to influence the work reported in this paper.
